# Regional Differences and Dynamic Evolution of Carbon Emission Intensity of Agriculture Production in China

**DOI:** 10.3390/ijerph17207541

**Published:** 2020-10-16

**Authors:** Jiaxing Pang, Hengji Li, Chengpeng Lu, Chenyu Lu, Xingpeng Chen

**Affiliations:** 1 College of Earth and Environmental Sciences, Lanzhou University, Tianshui South Road 222 #, Lanzhou 730000, China; lihengji@llas.ac.cn (H.L.); chenxp@lzu.edu.cn (X.C.); 2 Institute of County Economic Development, Lanzhou University, Tianshui South Road 222 #, Lanzhou 730000, China; lcp@lzu.edu.cn; 3 College of Geography and Environment Science, Northwest Normal University, Lanzhou 730070, China; lcy19810507@163.com

**Keywords:** agriculture carbon emission, Theil index, spatial correlation, spatial–temporal pattern

## Abstract

The study of the carbon emission intensity of agricultural production is of great significance for the formulation of a rational agricultural carbon reduction policy. This paper examines the regional differences, spatial–temporal pattern and dynamic evolution of the carbon emission intensity of agriculture production from 1991 to 2018 through the Theil index and spatial data analysis. The results are shown as follows: The overall differences in carbon emission intensity of agriculture production presents a slightly enlarging trend, while the inter-regional differences in carbon emissions intensity is decreasing, but the intra-regional difference of carbon emissions intensity presented an expanding trend. The difference in carbon emission intensity between the eastern and central regions is not obvious, and the difference in carbon emission intensity in the western region shows a fluctuating and increasing trend. The overall differences caused by intra-regional differences; the average annual contribution of intra-regional differences is 67.84%, of which the average annual contribution of western region differences is 64.24%. The carbon emission intensity of agricultural production in China shows a downward trend, with provinces with high carbon emission intensity remaining stable, while provinces with low intensity are expanding. The Global Moran’s I index indicates that China’s carbon emission intensity of agricultural production shows a clear trend of spatial aggregation. The agglomeration trend of high agricultural carbon emission remains stable, and the overall pattern of agricultural carbon emission intensity shows a pattern of increasing differentiation from east to west.

## 1. Introduction

Balanced economic development and environment protection have become hot topics around the world. With the rapid development of the global economy, global warming has brought great challenges to the sustainable development of human society. China has achieved remarkable economic growth and has become the world’s second largest economy, but it also has a large amount of carbon emissions [1]. China has become one of the largest greenhouse gas emitters [2,3], which accounts for nearly 30% of global emissions [4,5]. Therefore, China’s carbon emissions reduction will have a positive impact on global carbon emissions reduction. China has pledged to peak its carbon dioxide emissions by 2030 under the Paris Agreement, but study suggests that the peak may come earlier [6].

Although most of the carbon emissions come from industry and services, agricultural carbon emissions should not be underestimated. Greenhouse gas (GHG) emission from agricultural activities is one of the important parts of global GHG emissions [7,8]. Agriculture have become the second largest source of global greenhouse gas emissions, and the emissions are increasing at a fast speed of approximately 1% per annum [9,10]. Meanwhile, agriculture is the biggest source of anthropogenic non-CO_2_ emissions, being responsible for around 40% of total CH_4_, 60% of N_2_O and 20–35% of CO_2_ [11]. The agricultural sector is an important component of China’s national economy. China’s agricultural economy is developing rapidly; however, this rapid development has also led to a significant increase in carbon emissions. The major sources of agricultural carbon emissions are soil, intestinal fermentation of ruminants, biomass fuel burning, rice cultivation, and animal manure. Energy use in agricultural activities such as cultivation, land leveling, irrigation, farmland consolidation and fertilizing and herbicides production are also important sources of direct carbon emissions in agricultural sector [12]. Therefore, agricultural sector carbon emission reduction is an important link to improve the capability of agriculture to responses climate change; it is also a necessary choice to achieve economic growth, ecological environmental development, and sustainable agricultural development.

China’s agricultural activities produce a higher proportion of carbon than any other country [13], greenhouse gases from agricultural production accounting for 10–12% of global greenhouse gas emissions, compared to 16–17% in China [13,14,15,16], 17% of greenhouse gases, 50% of CH_4_, and 92% of N_2_O came from the agricultural sector in China [17,18,19]. China has decided to reduce its CO_2_ emission intensity by 40–50% by 2020 from the 2005 level [20]. The concept of green development has now become the goal of China’s socio-economic development.

This study focuses on the differences in the carbon emissions intensity of agricultural production in three major regions of China and the changes in the carbon emissions intensity of agricultural production at the provincial level. As a traditionally large agricultural country, due to different factors, there are great differences between different regions of China in the reduction of carbon emissions from agricultural economic development. Therefore, it has great significance to study the regional differences and spatial and temporal variations in the carbon emission intensity of Chinese agricultural to influence the formulation and implementation of carbon emissions reduction policies in the agricultural sector. However, before we can do that, we need to be clear about the source of carbon emissions, carbon emissions and carbon emissions intensity.

The aim of this paper is to further explore the temporal and spatial differentiation of carbon emissions from agricultural production in China. Firstly, this paper uses Theil index to quantitatively analyze the regional differences in the China’s agricultural production carbon emission intensity based on the regional differences and their causes. Secondly, the spatial measurement method is used to analyze the spatiotemporal change characteristics of carbon emission intensity of agricultural production in China. Finally, the spatial agglomeration of agricultural carbon emission intensity at provincial level is analyzed by using the Moran’ I index and hot and cold spot analysis. It also helps to put forward appropriate agricultural carbon emissions reduction strategies in different provinces of China.

## 2. Methodologies

### 2.1. Measuring Agricultural Carbon Emissions

The measurement of agricultural carbon emission is the basis for analysis for this paper. When producers engage in agricultural activities, they contribute to agricultural carbon emissions studied in this paper. According to the available data, the agricultural carbon emissions are mainly summarized into the following four aspects [21,22,23,24,25,26,27]: The first type of carbon emissions is caused by input to agricultural production, that is, agricultural irrigation, farmland ploughing and the use of fertilizers, pesticides, agricultural plastic sheeting and the consumption of diesel fuel from agricultural machinery; the second type of carbon emissions is caused by CH_4_ released during the growth of rice; the third type of carbon emissions is caused by N_2_O released from the soil during crop cultivation; the fourth type of carbon emissions is caused by CH_4_ and N_2_O released from animal husbandry.

Due to emissions of CH_4_ and N_2_O, we need to convert CH_4_ and N_2_O into carbon emissions. According to the IPCC’s Fifth Assessment Report, the greenhouse effect induced by 1 ton of CH4 is equivalent to that produced by 6.8182 tons of carbon and the greenhouse effect caused by 1 ton of N_2_O is equivalent to that produced by 81.2727 tons of carbon). The formula for calculating the agricultural carbon emissions is as follows [27]:

(1) $$C=\overset{n}{\underset{i=1}{\sum }}T_{i}\times \delta_{i}$$

where *C* denotes the total agricultural carbon emissions, $T_{i}$ is the agricultural carbon emissions of different source, $\delta_{i}$ is the coefficient of different agricultural carbon source.

### 2.2. The Theil Index

The Theil index is a statistic used to measure economic inequality and other economic phenomena. Now, it has also been used to measure the imbalance of regional development. The Theil index can decompose the regional overall differences *(T)* into two parts: Inter-regional differences *(Tbr)* and intra-regional differences between different provinces *(Twr)* to analyze their contribution to the total differences and the main sources of overall differences [28].

Thus, this paper uses the Theil index to calculate the regional differences in carbon emissions from China’s agricultural production. The Theil index is defined as follows [29,30,31]:

(2) $$T=\overset{n}{\underset{i=1}{\sum }}x_{i}\left[\overset{m}{\underset{j=1}{\sum }}x_{ij}lnd_{ij}\right]+\overset{n}{\underset{i=1}{\sum }}x_{ij}lnd_{i}=T_{br}+T_{wr}$$

In this formula, *n* and *m* represent the number of regions and the number of provinces within the region, respectively; *x_i_* represents the proportion of carbon emissions from agricultural production in region *i* to total carbon emissions from agricultural production in China; *x_ij_* represents the proportion of carbon emissions from agricultural production in province *j* within region *i* to total carbon emissions from agricultural production in China; *d_i_* represents the ratio of the intensity of carbon emissions from agricultural production in region *i* to total carbon emissions intensity from agricultural production in China; *d_ij_* represents the ratio of the intensity of carbon emissions from agricultural production in province *j* within region *i* to total carbon emissions from agricultural production in China. Meanwhile, in order to calculate the contribution of different differences from the overall difference, we used the quotient expression contribution of each difference index and the overall difference index to calculate [32].

### 2.3. The Global Spatial Auto-Correlation

Spatial autocorrelation is a spatial data analysis method that studies whether the observed values at one location in space are correlated with the observed value at its adjacent positions, and can reveal the regional structural forms of spatial variables. Spatial autocorrelation analysis can be divided into global spatial autocorrelation and local spatial autocorrelation [33].

Global Spatial autocorrelation is a description of the spatial characteristics of attribute values in the whole region [34,35]. The global spatial autocorrelation is shown as follows:

(3) $$I=\frac{n\sum_{i=1}^{n}\sum_{j=1}^{n}W_{ij}\left(x_{i}-\bar{x}\right)\left(x_{j}-\bar{x}\right)}{\sum_{i=1}^{n}\sum_{j=1}^{n}W_{ij}\sum_{i=1}^{n}{\left(x_{j}-\bar{x}\right)}^{2}}$$

*I* stands for Global Moran’I index; *n* stands for the number of provinces; *x_i_* and *x_j_* represent carbon emissions intensity of agricultural production in provinces *i* and *j*, respectively; $\bar{x}$ stands for the average value of carbon emissions intensity from agricultural production of each province; *W_ij_* stands for space weights of provinces *i* and *j*.

### 2.4. Hot and Cold Spot Analysis

The Global spatial correlation analysis can reflect the over spatial characteristics, but cannot analyze the local spatial characteristics and determine the specific location of the clustering. Getis-Ord Gi* can measure the density of high value (hot spot) and low value (cold spot) for a specified study area. The formula is as follows:

(4) $$G_{i}^{*}\left(d\right)=\frac{\sum_{j}^{n}W_{ij}x_{j}}{\sum_{i}^{n}x_{i}}.$$

The corresponding standardized statistics for the index $G_{i}^{*}\left(d\right)$ are $Z\left(G_{i}^{*}\right)$:

(5) $$Z\left(G_{i}^{*}\right)=\frac{G_{i}^{*}\left(d\right)-E\left(G_{i}^{*}\right)}{\sqrt{VAR\left(G_{i}^{*}\right)}}.$$

In this formula, *n* stands for the number of provinces, *Wij* stands for space weights of provinces *i* and *j*, *x_i_* represent carbon emissions intensity of agricultural production in provinces *i*,$E\left(G_{i}^{*}\right)$ and $\sqrt{VAR\left(G_{i}^{*}\right)}$ stands for the expectation and variance of the $G_{i}^{*}\left(d\right)$. $Z\left(G_{i}^{*}\right)$ positive and significant indication that surrounding area of province *i* is a high-value agglomeration area, that is, hot spot area; otherwise, it is a cold spot area.

### 2.5. Data Source

The research area for this study is mainland China, except for Hong Kong, Macao, and Taiwan province. This paper focuses on 1991–2018. All the data in this paper were collected from the China Rural Statistical Yearbook (1992–2019) [36]. The variables used in this study include agricultural carbon emissions and agricultural economy. Agricultural carbon emissions data have been calculated, and the agricultural economy data are expressed by the added value of agriculture, forestry, animal husbandry, and fishery.

## 3. Regional Differences in China’s Carbon Emissions from Agricultural Production

**Figure 1:**
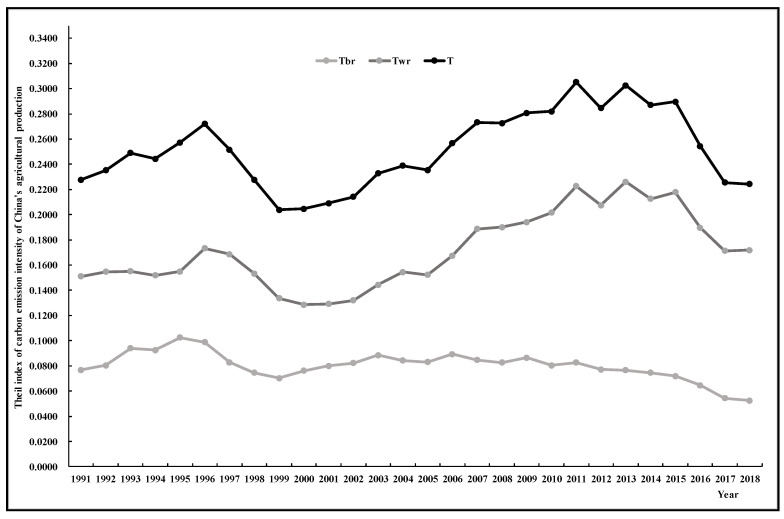
Theil index of carbon emission intensity of agriculture in China from 1991 to 2018.

**Figure 2:**
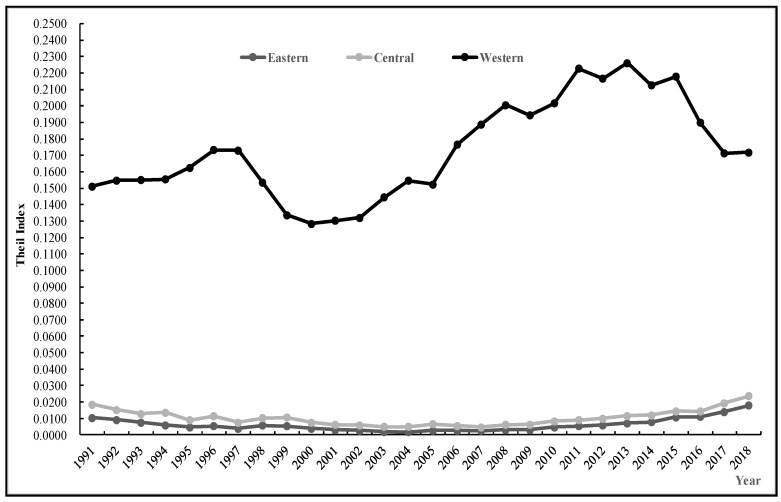
Chang trend of regional Theil index.

According to formula, the Theil index of carbon emission intensity of agricultural production in China can be calculated and decomposed, the regional overall differences *(T)* can be decomposed into two parts: Inter-regional differences *(Tbr)* and intra-regional differences between different provinces *(Twr)*, the intra-regional differences *(Twr)* can be decomposed into intra-regional differences in eastern, central, and western regions. The results are shown in Figure 1 and Figure 2, Table 1 and Table 2.

### 3.1. Overall Difference Analysis

Based on the calculated overall Theil index, this paper analyzes the regional and interregional differences in carbon emission intensity of agricultural production in China. Figure 1 shows the “up–down–up–down” trend across the Theil index, the overall Theil index has demonstrated a cyclical trend in the whole study period, which showed an *M*-type change, but the overall trend was upward. The overall Theil index increased from 0.2278 in 1991 to 0.2721 in 1996, decreased to 0.2039 in 1999, rose again to 0.3053 in 2011, and finally dropped again to 0.2243 in 2018. This indicates that the overall regional difference in the carbon emission intensity of agricultural production in China shows a dynamic trend of “widening–narrowing–widening–narrowing”. The overall difference widens slightly, with the Theil index growing at an average annual rate of 0.14%. In addition, the *Tbr* shows an *M*-type variation, but the trend demonstrates a weak decrease, with an average annual decline rate of 1.1%, which indicates that the inter-regional difference in carbon emission intensity from agricultural production among the eastern, central, and western regions was narrowing.

### 3.2. Analysis of Intra-Regional Difference

In order to reveal the difference in carbon emission intensity of agricultural production in eastern, central and Western China, the intra-regional differences in carbon emission intensity of agricultural production in China are analyzed by using the *Twr*. As can be seen from Table 1 and Figure 1, there was a similar trend between the *Twr* and the overall *T* during the study period, indicating that the differences in the three regions underwent a dynamic change process of “widening–narrowing–widening–narrowing”. However, the overall trend was slightly upward, with an average annual growth rate of 0.74%.

According to Table 2 and Figure 2, the Theil index for the western region is significantly higher than that for the eastern and the central regions, and the Theil index for eastern region is slightly higher than that for central region throughout the study process. The results show that the difference of agricultural carbon intensity in the western provinces is larger than that in eastern and central provinces, and the difference of agricultural production carbon intensity in the central provinces is the smallest. In the western region, the differences of agricultural carbon intensity among the provinces are similar to the differences of overall agricultural production carbon intensity, while the differences in carbon emission intensity of agricultural production among provinces in the eastern region showed a U-shaped trend. The difference of carbon emission coefficient of agricultural production among provinces in central China showed a fluctuating but overall decreasing trend.

### 3.3. Cause Analysis of Regional Differences

By comparing the contribution proportion of the Theil index to China’s agricultural regional carbon emission coefficient between 1991 and 2018, as shown in Table 3, the reasons for the regional differences can be better understood.

Through the analysis of Table 3, the results show that the regional differences of carbon emission intensity of agricultural production in China are mainly dependent on the intra-regional differences during the whole study period. The contribution of the intra-regional differences increased from 66.30% in 1991 to 76.63% in 2018, with an average annual growth rate of 0.58% and an average contribution rate of 67.84%. The contribution of inter-regional differences showed a fluctuating downward trend, from 33.68% in 1991 to 23.37% in 2018, with an average annual decline rate of 1.21% and an average contribution rate of 32.16%. This indicates that the overall difference in the carbon emission intensity of agricultural production is increasingly dependent on the intra-regional difference, and the increase in the contribution of the difference within the region is mainly due to the gradual expansion of the difference in the carbon emission intensity of agricultural production in western China. The differential contribution of the intensity of carbon emissions from agricultural production between provinces in the western region increased from 58.18% in 1991 to 66.10% in 2017, with an average contribution rate of 64.24% and an average annual growth rate of 0.54%.

## 4. Spatial Pattern Evolution

### 4.1. Spatial and Temporal Sequence Analysis of Agricultural Carbon Emission Intensity

**Figure 3:**
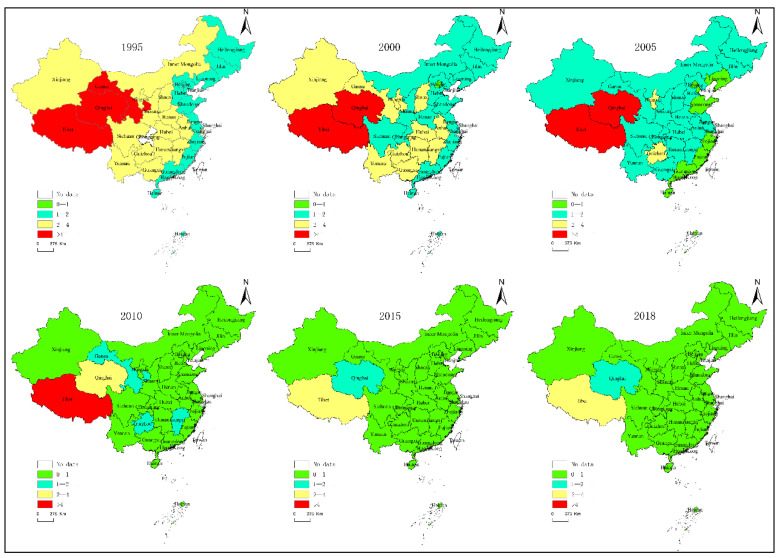
Spatial pattern of the carbon emission intensity of China’s agricultural production.

The carbon emission intensity of agricultural production in China is divided into four levels. ArcGIS is used to visualize the spatial pattern of the carbon emission intensity of China’s agricultural production during the study process. Overall, the spatial distribution of the other regions of carbon emission intensity changed greatly during the study period except for the high intensity provincial region, and the higher intensity region showed a contraction trend, while the lower intensity region showed an expansion trend, indicating that the overall agricultural carbon emission intensity showed a decreasing trend in China. Figure 3 shows that Tibet and Qinghai province keep a relatively high emission intensity level throughout the study period. Before 2005, carbon emission intensity of agricultural production showed a step-like distribution characteristic, for the number was higher in the west and lower in the east. After 2005, the carbon emission intensity of agricultural production of all provinces entered a low period, which also indicates that carbon emission intensity of China’s agricultural production indicates an overall downward trend, especially in the central and eastern regions.

**Figure 4:**
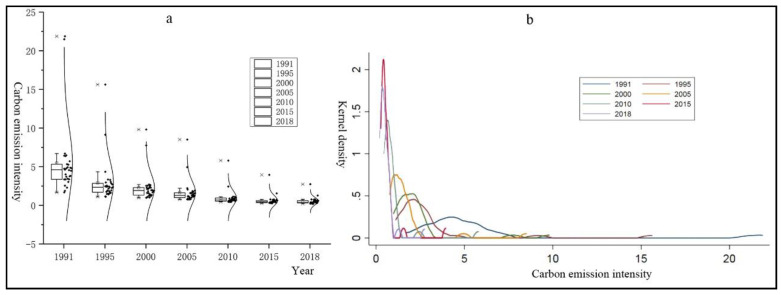
(**a**) Box-plot of carbon emission intensity of China’s agricultural production; (**b**) The Kernel density estimation of carbon emission intensity of China’s agricultural production.

According to Figure 4a, the average carbon emission intensity of China’s agricultural production has been gradually decreasing over the past 28 years, and the differences among provinces have been shrinking as well, there is a converging trend among the provinces. In order to have a further understanding of the distribution of agricultural production carbon emission intensity, Figure 4b can serve as a reference, for it shows the kernel density estimation for 1991, 1995, 2000, 2005, 2010, 2015, and 2018. The overall trend of the distribution of the kernel density curve changes, with the peak shifting to the left and turning into “double peak”, indicating that the differences between provinces is narrowing while the distribution of carbon emission intensity shows polarization phenomenon; the tail of the kernel density curve becomes shorter, indicating that the number of provinces with low carbon emission intensity is increasing.

### 4.2. Global Pattern Evolution

**Figure 5:**
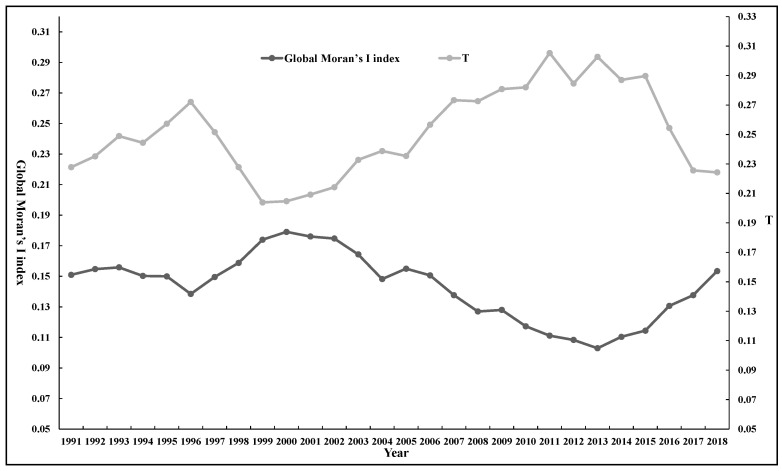
Global Moran’s I index and overall Theil index trends.

The Global Moran’s I index of China’s carbon emission intensity of agricultural production and the statistical *Z*-value from 1991 to 2018 (Table 4 and Figure 5) indicate that the Global Moran’s I index is significantly positive between 0.103 and 0.179. The Global Moran’s I index passed the significance *Z* statistical test, which indicates that China’s carbon emission intensity of agricultural production exhibits a significant spatial aggregated trend. Meanwhile, Global Moran’s I showed an down–up–down–up trend, which reveals the aggregation is continuously evolving over the time, which implies evolution of aggregation among the regions with similar carbon emission intensity of agricultural production in China’s as well. Figure 5 showed that there is a strong correlation between the overall difference of carbon emission intensity in agricultural production and the global spatial autocorrelation index. During the whole period, the correlation coefficient of the two indicators is −0.92585, which fully shows that the deepening of the overall difference degree of carbon emission intensity will lead to a weakening of the geographic pattern of the agglomeration of carbon emission intensity, which will thus reducing the global spatial autocorrelation degree of carbon emissions intensity.

### 4.3. Hot and Cold Spot Analysis

**Figure 6:**
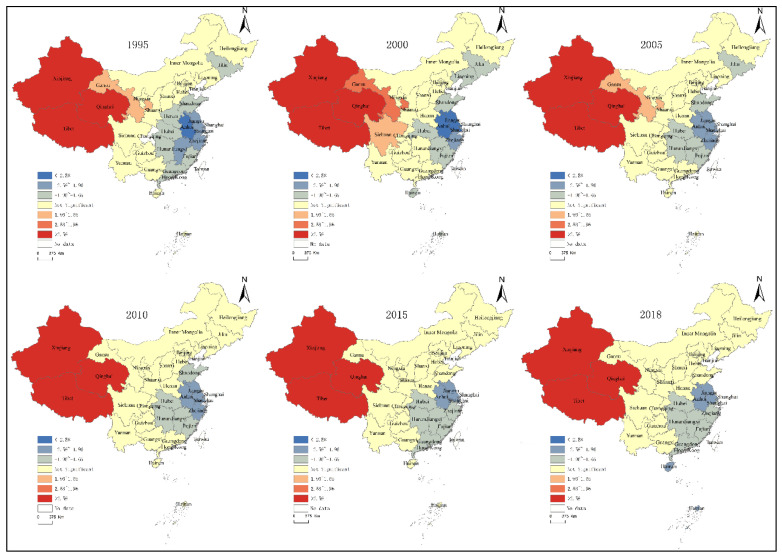
Cold and hot spots of carbon emission intensity of China’s agricultural production.

The hot spots–cold spots identified by ArcGIS local spatial correlation index Getis-Ord Gi*, are spatially clustered with statistically significant high (low) values. The results are shown in Figure 6. The agglomeration trend of high intensity and low intensity of agricultural carbon emission remains stable, and the agricultural carbon emission intensity overall presents that the pattern of increasing differentiation from east to west. From 1991 to 2018, the evolution of hot spot–cold spot of carbon emissions intensity of agricultural production has the following features: The spatial distribution of carbon emission intensity and the hot cold spot distribution of carbon emission intensity of China’s agricultural production have spatial convergence. The core hot spots are mainly located in Western China like Xinjiang, Tibet, and Qinghai, which are important domestic animal production areas. The carbon emissions from animal husbandry in these three provinces account for more than 70% of the total agricultural carbon emissions in every province. However, the development of agricultural economy is relatively slow; the cold spots are mainly in the Yangtze River Delta, where the agricultural development level is high and the carbon emission of agricultural production is relatively low. No significant change can be observed in the pattern of the hot spot–cold spot agglomeration of agricultural carbon intensity in China. Inner Mongolia, Heilongjiang, Ningxia, Shanxi, Shaanxi, Hebei, Beijing, Tianjin, Chongqing, Yunnan, Guizhou, and Guangxi have all become transitional regions. The core hot spots remain unchanged. Among the sub-hotspots and marginal hotspots, only Gansu province has experienced the change of marginal hot-spot area–sub-hotspot area–marginal hot-spot area–transition zone, while Sichuan has experienced the change of transition zone–marginal hot spot zone–transition zone. Although the sub-cold spot area and marginal cold spot area changed in some provinces, the overall pattern did not change significantly, and Henan, Shandong and Jilin provinces changed from marginal cold spots to transitional areas.

## 5. Conclusions

Based on the calculation of the carbon emissions intensity of agricultural production from 1991 to 2018 in China, this paper analyzes and discusses the regional differences and spatial and temporal pattern characteristics of the carbon emission intensity of agricultural production in China. The results show that:

The overall Theil index changed from 0.2278 in 1991 to 0.2243 in 2018, which indicates that the overall regional difference in the carbon emission intensity of agricultural production in China shows a dynamic trend, but the overall difference widens slightly. In addition, the *Tbr* showed a weak downward trend, which indicates that the inter-regional differences among the eastern, central, and western regions was narrowing. The *Twr* showed an upward trend, which indicates that the intra-regional differences were widening, as the differences in carbon emission intensity in the western region shows a fluctuating upward trend, the difference of agricultural carbon intensity in the western provinces is larger than that in eastern and central provinces, and the difference of agricultural production carbon intensity in the central provinces is the smallest. The overall differences were mainly caused by intra-regional differences, with the average annual contribution of intra-regional differences being 67.84%, of which the average annual contribution of western region differences was 64.24%.The carbon emission intensity of agricultural production in China showed a downward trend, the distribution of carbon emission intensity shows polarization phenomenon; with provinces with high carbon emission intensity remaining stable, while the number of provinces with low carbon emission intensity is increasing. The Global Moran’s I index is significantly positive, which indicates that China’s carbon emission intensity of agricultural production exhibits a significant spatial aggregated trend. There is a strong correlation between the T and the Global Moran’s I index, with a correlation coefficient of −0.92585, which fully indicates that the deepening of the overall difference degree in carbon emission intensity will lead to the weakening of the geographical pattern of the agglomeration of carbon emission intensity. The agglomeration trend of high and low intensity of agricultural carbon emission remains stable, with hot spots concentrated in the west and the cold spots in the east, and the overall carbon emission intensity of agricultural production presents that the pattern of increasing differentiation from east to west.

Further research on the regional difference and spatial agglomeration of agricultural carbon emissions intensity through the regional difference analysis model could provide ideas of the regional emission reduction of agricultural carbon emission.

## Figures and Tables

**Table 1:** Theil index of carbon emissions intensity of China’s agricultural production.

Year	Tbr	Twr	T
1991	0.0767	0.1510	0.2278
1992	0.0804	0.1547	0.2352
1994	0.0926	0.1518	0.2444
1995	0.1025	0.1548	0.2573
1996	0.0989	0.1733	0.2721
1997	0.0829	0.1687	0.2516
1998	0.0745	0.1533	0.2278
1999	0.0702	0.1337	0.2039
2000	0.0762	0.1285	0.2047
2001	0.0801	0.1291	0.2092
2002	0.0822	0.1321	0.2142
2003	0.0885	0.1444	0.2328
2004	0.0843	0.1546	0.2388
2005	0.0830	0.1523	0.2354
2006	0.0892	0.1674	0.2566
2007	0.0846	0.1888	0.2733
2008	0.0825	0.1901	0.2726
2009	0.0865	0.1942	0.2808
2010	0.0803	0.2017	0.2820
2011	0.0826	0.2227	0.3053
2012	0.0771	0.2075	0.2846
2013	0.0765	0.2262	0.3027
2014	0.0744	0.2126	0.2870
2015	0.0718	0.2179	0.2897
2016	0.0647	0.1898	0.2544
2017	0.0544	0.1712	0.2256
2018	0.0524	0.1719	0.2243

**Table 2:** The intra-regional difference of carbon emissions intensity of China’s agricultural production.

Year	Eastern	Central	Western
1991	0.0105	0.0080	0.1325
1992	0.0092	0.0060	0.1395
1993	0.0077	0.0052	0.1421
1994	0.0059	0.0078	0.1416
1995	0.0048	0.0042	0.1534
1996	0.0054	0.0061	0.1618
1997	0.0039	0.0037	0.1654
1998	0.0057	0.0045	0.1430
1999	0.0054	0.0052	0.1231
2000	0.0040	0.0035	0.1210
2001	0.0032	0.0030	0.1240
2002	0.0026	0.0033	0.1261
2003	0.0020	0.0030	0.1394
2004	0.0018	0.0033	0.1495
2005	0.0027	0.0039	0.1457
2006	0.0028	0.0029	0.1707
2007	0.0026	0.0021	0.1840
2008	0.0031	0.0031	0.1943
2009	0.0031	0.0032	0.1879
2010	0.0048	0.0037	0.1932
2011	0.0054	0.0037	0.2136
2012	0.0060	0.0039	0.2066
2013	0.0071	0.0045	0.2146
2014	0.0078	0.0042	0.2005
2015	0.0108	0.0038	0.2033
2016	0.0110	0.0032	0.1756
2017	0.0140	0.0053	0.1519
2018	0.0180	0.0056	0.1483

**Table 3:** Contribution rate of Theil index of agricultural production carbon emission intensity in China from 1991 to 2018.

Year	Intra-Regional (%)			Inter-Regional (%)	Intra-Regional (%)
	Eastern	Central	Western		
1991	4.62	3.51	58.18	33.68	66.30
1992	3.92	2.54	59.34	34.20	65.80
1993	3.08	2.10	57.09	37.74	62.26
1994	2.41	3.20	57.95	37.88	62.11
1995	1.88	1.62	59.61	39.85	60.16
1996	1.97	2.23	59.47	36.33	63.67
1997	1.56	1.49	65.73	32.95	67.05
1998	2.50	2.00	62.78	32.72	67.28
1999	2.65	2.55	60.38	34.43	65.57
2000	1.96	1.71	59.09	37.23	62.77
2001	1.54	1.45	59.29	38.28	61.71
2002	1.23	1.55	58.88	38.35	61.65
2003	0.86	1.29	59.86	38.00	62.01
2004	0.74	1.36	62.61	35.28	64.72
2005	1.13	1.67	61.91	35.28	64.72
2006	1.08	1.11	66.54	34.77	65.24
2007	0.95	0.78	67.33	30.94	69.06
2008	1.14	1.13	71.28	30.27	69.74
2009	1.12	1.15	66.92	30.82	69.18
2010	1.69	1.32	68.50	28.48	71.52
2011	1.75	1.21	69.98	27.06	72.94
2012	2.12	1.38	72.58	27.08	72.91
2013	2.34	1.48	70.90	25.28	74.72
2014	2.73	1.48	69.86	25.93	74.07
2015	3.72	1.30	70.19	24.79	75.21
2016	4.32	1.26	69.01	25.41	74.59
2017	6.20	2.35	67.35	24.10	75.90
2018	8.03	2.51	66.10	23.37	76.63

**Table 4:** Global Moran’s I index of China’s carbon emission intensity of agricultural production from 1991 to 2018.

Year	1991	1992	1993	1994	1995	1996	1997
MI	0.151 ***	0.155 ***	0.156 ***	0.150 ***	0.150 ***	0.138 ***	0.150 ***
Z	2.838	2.925	3.000	2.976	3.102	2.882	3.104
Year	1998	1999	2000	2001	2002	2003	2004
MI	0.159 ***	0.174 ***	0.179 ***	0.176 ***	0.175 ***	0.164 ***	0.148 ***
Z	3.165	3.329	3.342	3.349	3.345	3.220	3.108
Year	2005	2006	2007	2008	2009	2010	2011
MI	0.155 ***	0.151 ***	0.138 ***	0.127 ***	0.128 ***	0.117 ***	0.111 ***
Z	3.182	3.133	3.063	3.024	3.003	2.885	2.866
Year	2012	2013	2014	2015	2016	2017	2018
MI	0.108 ***	0.103 ***	0.110 ***	0.114 ***	0.131 ***	0.138 ***	0.153 ***
Z	2.838	2.789	2.860	2.869	2.960	2.859	3.134

**Notes**: *** Denotes significance at the 0.01 level.
